# Supplementary material for: Cross-cultural adaptation of the 4-Habits Coding Scheme into French to assess physician communication skills
Source: PLoS One. 2020 Apr 16;15(4):e0230672. doi: 10.1371/journal.pone.0230672 (PMC7161987; doi:10.1371/journal.pone.0230672)
Supplement: S1 Appendix — (DOCX) [file pone.0230672.s001.docx]

**Appendix 1. Cross-cultural adaptation of the 4-HCS scale into French.**

| Système de Codage des Quatre habitudes  *Université Grenoble Alpes. Avec le soutien de Fondation MACSF.*  Consignes : Notez chacun des items ci-dessous en utilisant les niveaux 1, 3 ou 5. Si vous pensez que la compétence se situe à un niveau intermédiaire, vous pouvez utiliser 2 ou 4. | | |
| --- | --- | --- |
| Critères d’évaluation | Degré d’accord | |
| *Habitude 1 : S’investir dès le début (/30)* | | |
| A. Montre de la familiarité avec le patient | | |
| Le clinicien a besoin de se reporter systématiquement au dossier du patient pour s’y familiariser ou ne fait pas de rapprochement entre la consultation en cours et les antécédents ou le dossier du patient (ou n’a même pas le dossier) | 1 |  |
|  | (2) |  |
| Le clinicien fait référence à des consultations précédentes ou aux antécédents du patient, mais ne semble pas bien connaître le sujet | 3 |  |
|  | (4) |  |
| Le clinicien connaît bien les antécédents/le dossier médical du patient (par ex., fait référence à des examens récemment pratiqués ou à des informations notées lors de consultations précédentes) | 5 |  |
| B. Accueille les patients chaleureusement | | |
| L'accueil du patient est rapide, impersonnel ou inexistant. | 1 |  |
|  | (2) |  |
| L'accueil du clinicien montre qu'il reconnaît le patient, mais l'accueil n'est ni très chaleureux ni personnalisé. | 3 |  |
|  | (4) |  |
| Le patient est accueilli de manière personnelle et chaleureuse (par ex., le clinicien lui demande comment il souhaite qu'on s'adresse à lui, l'appelle par son nom). | 5 |  |
| C. Fait du bavardage | | |
| Le clinicien va droit au but sans aucune tentative de bavardage (ou coupe la parole au patient sèchement et brutalement, ou s'il montre un quelconque intérêt, ce n'est qu'un intérêt superficiel plus tard au cours de la consultation). | 1 |  |
|  | (2) |  |
| Le clinicien fait une rapide tentative de bavardage (ne montre pas grand intérêt, met rapidement fin à la conversation avant de poursuivre). | 3 |  |
|  | (4) |  |
| Le clinicien fait des commentaires d'ordre non médical afin de mettre le patient à l’aise. | 5 |  |
| D. Utilise principalement des questions ouvertes | | |
| Le clinicien essaie d'identifier le(s) problème(s), principalement à l'aide de questions fermées (conversation saccadée). | 1 |  |
|  | (2) |  |
| Le clinicien associe des questions ouvertes et fermées pour tenter d'identifier le(s) problème(s) (par ex. il commence par une question ouverte, puis revient rapidement à des questions fermées). | 3 |  |
|  | (4) |  |
| Le clinicien essaie d'identifier le(s) problème(s) principalement à l'aide de questions ouvertes (formule ses questions de façon à ce que le patient raconte sa propre histoire, en essayant de ne pas l'interrompre et avec un minimum de questions fermées). | 5 |  |
| E. Encourage l’expression des inquiétudes du patient | | |
| Le clinicien interrompt le patient et lui coupe la parole lorsqu'il essaie d'approfondir sa réponse (n'est clairement pas très intéressé). | 1 |  |
|  | (2) |  |
| Le clinicien n'interrompt pas le patient, mais ne cherche pas à en savoir davantage (écoute, mais n'encourage pas le patient à approfondir ou à développer). | 3 |  |
|  | (4) |  |
| Le clinicien encourage le patient à approfondir la conversation et à exprimer ses inquiétudes (par ex., à l'aide de formules incitatives comme « Ah ? », « Dites-m'en plus », « Continuez »). | 5 |  |
| F. Suscite toutes les préoccupations | | |
| Le clinicien s'intéresse immédiatement à la première préoccupation du patient sans tenter de découvrir si le patient a d'autres éventuelles préoccupations. | 1 |  |
|  | (2) |  |
| Le clinicien fait référence à d'autres sources d'inquiétudes possibles ou demande rapidement au patient s'il a d'autres inquiétudes, avant de s'intéresser au premier problème évoqué par le patient, ou établit un ordre du jour au fur et à mesure de la consultation. | 3 |  |
|  | (4) |  |
| Le clinicien tente de recueillir l'ensemble des préoccupations du patient, en établissant un ordre du jour dès le début de la consultation (le clinicien va bien au-delà de la première préoccupation énoncée). | 5 |  |
| *Habitude 2 : Obtenir le point de vue du patient (/15)* | | |
| A. Intéressé par la compréhension du problème du patient | | |
| Le clinicien ne cherche pas à comprendre/ne s'intéresse pas au point de vue du patient. | 1 |  |
|  | (2) |  |
| Le clinicien ne s'intéresse que brièvement ou superficiellement à la façon dont le patient comprend le problème. | 3 |  |
|  | (4) |  |
| Le clinicien fait preuve d'un grand intérêt quant à la façon dont le patient comprend le problème (par ex., il lui demande ce que ses symptômes signifient pour lui). | 5 |  |
| B. Demande les attentes du patient pour la consultation | | |
| Le clinicien n'essaie aucunement de déterminer (ne s'intéresse pas à) ce que le patient attend de la consultation. | 1 |  |
|  | (2) |  |
| Le clinicien s'intéresse brièvement à ce que le patient attend de la consultation, mais passe rapidement à la suite. | 3 |  |
|  | (4) |  |
| Le clinicien demande (ou réagit avec intérêt à) ce que le patient attend de la consultation (par ex., s'il a des attentes générales ou spécifiques, telles qu'une ordonnance, l'orientation vers un spécialiste). | 5 |  |
| C. Montre de l’intérêt à propos de l’impact sur la vie quotidienne | | |
| Le clinicien n'essaie aucunement de déterminer/ne montre aucun intérêt quant à l'impact que le problème peut avoir sur la vie quotidienne du patient. | 1 |  |
|  | (2) |  |
| Le clinicien cherche brièvement à déterminer/ne montre qu’un intérêt limité quant à l'impact que le problème peut avoir sur la vie quotidienne du patient. | 3 |  |
|  | (4) |  |
| Le clinicien cherche à déterminer précisément/s'intéresse réellement à l'impact que le problème peut avoir sur la vie quotidienne du patient (au niveau professionnel, familial, sur les activités quotidiennes). | 5 |  |
| *Habitude 3 : Faire preuve d’empathie (/20)* | | |
| A. Encourage l’expression des émotions | | |
| Le clinicien ne fait preuve d'aucun intérêt quant à l'état émotionnel du patient et/ou décourage ou interrompt le patient quand celui-ci exprime des émotions (signale de façon verbale ou non verbale que le patient ne devrait pas exprimer d'émotions). | 1 |  |
|  | (2) |  |
| Le clinicien s'intéresse relativement peu aux émotions du patient, ne l'encourage pas à les exprimer, ou laisse le patient montrer ses émotions, mais l'encourage de façon évidente ou subtile à ne pas s'y attarder. | 3 |  |
|  | (4) |  |
| Le clinicien est réceptif aux émotions exprimées par le patient et l'encourage ouvertement (par ex., il incite le patient à poursuivre ou marque des pauses, montre, de façon verbale ou non, qu'il peut exprimer ses émotions). | 5 |  |
| B. Accepte/comprend les sentiments du patient | | |
| Le clinicien n'essaie nullement de montrer qu'il valide les sentiments du patient, voire même les dénigre ou les conteste (par ex., « C'est ridicule de s'inquiéter autant de... »). | 1 |  |
|  | (2) |  |
| Le clinicien prend brièvement acte des sentiments du patient, mais ne fait aucun effort pour montrer qu'il reconnaît leur validité/leur bien-fondé. | 3 |  |
|  | (4) |  |
| Le clinicien fait des commentaires qui montrent clairement qu'il reconnaît la validité/le bien-fondé des sentiments du patient (par ex., « Je me sentirais comme vous à votre place », « Je comprends que ça vous puisse inquiéter »). | 5 |  |
| C. Aide à identifier/nommer les sentiments | | |
| Le clinicien n'essaie aucunement d'identifier les sentiments du patient. | 1 |  |
|  | (2) |  |
| Le clinicien fait brièvement référence aux sentiments du patient, mais ne cherche pas vraiment à approfondir en les identifiant ou en les nommant. | 3 |  |
|  | (4) |  |
| Le clinicien essaie manifestement d'explorer davantage les sentiments du patient en les identifiant ou en les nommant (par ex., « Qu'est-ce que cela vous fait ? », « Vous me semblez très inquiet au sujet de... »). | 5 |  |
| D. Fait preuve d’un comportement non verbal efficace | | |
| Le comportement non verbal du clinicien montre qu'il ne s'intéresse pas, ne se préoccupe pas et/ou marque une certaine distance avec le patient (par ex., peu ou pas de contact du regard, orientation du corps ou utilisation de l'espace inadéquate, ton de voix qui dénote l'ennui). | 1 |  |
|  | (2) |  |
| Le comportement non verbal du clinicien ne témoigne ni réel intérêt ni désintérêt (ou son comportement change au cours de la consultation). | 3 |  |
|  | (4) |  |
| Le comportement non verbal du clinicien témoigne d'un grand intérêt, il se montre concerné et il établit une certaine proximité avec le patient (par ex., en le regardant dans les yeux, au travers du ton de la voix et de l'orientation du corps) pendant toute la consultation. | 5 |  |
| *Habitude 4 : S’investir jusqu’à la fin (/50)* | | |
| A. Énonce les informations/le diagnostic en fonction des préoccupations du patient | | |
| Le clinicien énonce le diagnostic et les informations en des termes qui correspondent aux références du médecin plutôt que de s'adapter au patient | 1 |  |
|  | (2) |  |
| Le clinicien essaie sommairement d'énoncer le diagnostic et les informations en termes de préoccupations du patient. | 3 |  |
|  | (4) |  |
| Le clinicien énonce le diagnostic et d'autres informations pertinentes d'une façon qui fait écho aux préoccupations initiales du patient. | 5 |  |
| B. Marque des pauses permettant l’intégration de l’information | | |
| Le clinicien donne les informations et poursuit rapidement, sans donner au patient la possibilité de réagir (donnant l'impression que le patient ne se rappellera pas ou n’intégrera pas complètement toutes les informations). | 1 |  |
|  | (2) |  |
| Le clinicien marque une courte pause pour laisser au patient le temps de réagir, puis passe rapidement à autre chose (donnant l’impression que le patient n'a peut-être pas complètement intégré les informations). | 3 |  |
|  | (4) |  |
| Le clinicien marque une pause après avoir donné les informations au patient afin de lui laisser le temps de réagir et de de les intégrer. | 5 |  |
| C. Explique clairement/sans jargon médical | | |
| Les informations sont présentées en termes techniques ou inaccessibles au patient (indiquant que le patient n'a probablement pas entièrement ou correctement compris ces informations). | 1 |  |
|  | (2) |  |
| Les informations contiennent du jargon et sont quelque peu difficiles à comprendre | 3 |  |
|  | (4) |  |
| Les informations sont claires et contiennent peu ou pas de jargon | 5 |  |
| D. Explique la nécessité des examens et traitements | | |
| Le clinicien propose de prescrire/prescrit des examens et un traitement, avec peu ou pas d'explication | 1 |  |
|  | (2) |  |
| Le clinicien n'explique que brièvement la raison des examens et du traitement | 3 |  |
|  | (4) |  |
| Le clinicien explique de façon complète et claire la nécessité des examens et des traitements en cours, passés ou futurs, de façon à ce que le patient comprenne leur importance pour poser le diagnostic et décider du traitement | 5 |  |
| E. Vérifie efficacement la compréhension | | |
| Le clinicien ne fait aucun effort pour déterminer si le patient a compris ce qui a été dit | 1 |  |
|  | (2) |  |
| Le clinicien vérifie de façon rapide et insuffisante si le patient a compris | 3 |  |
|  | (4) |  |
| Le clinicien s'assure que le patient a compris | 5 |  |
| F. Encourage la participation à la prise de décision | | |
| Le clinicien ne montre aucun intérêt à impliquer le patient ou décourage manifestement/ignore ses tentatives pour participer aux prises de décision | 1 |  |
|  | (2) |  |
| Le clinicien montre peu d'intérêt à impliquer le patient dans la prise de décision, ou se montre relativement peu enthousiaste lorsque le patient essaie de s'impliquer | 3 |  |
|  | (4) |  |
| Le clinicien encourage clairement le patient et l'invite à participer activement à la prise de décision | 5 |  |
| G. Vérifie l’acceptation du plan thérapeutique | | |
| Le clinicien recommande le traitement sans réellement essayer d'obtenir l’acceptation du patient (volonté ou susceptibilité de suivre le plan thérapeutique). | 1 |  |
|  | (2) |  |
| Le clinicien tente brièvement de déterminer si le patient accepte le plan thérapeutique et passe rapidement à autre chose | 3 |  |
|  | (4) |  |
| Le clinicien vérifie que le patient accepte le plan thérapeutique, et montre qu'il est prêt à négocier, si besoin | 5 |  |
| H. Explore les obstacles à sa mise en œuvre | | |
| Le clinicien ne mentionne pas les éventuels obstacles à la mise en œuvre du plan thérapeutique | 1 |  |
|  | (2) |  |
| Le clinicien présente brièvement les potentiels obstacles à la mise en œuvre du plan thérapeutique. | 3 |  |
|  | (4) |  |
| Le clinicien présente de façon exhaustive tous les obstacles qui pourraient empêcher la mise en œuvre du plan thérapeutique | 5 |  |
| I. Encourage des questions supplémentaires | | |
| Le clinicien n'encourage aucunement le patient à poser des questions supplémentaires ou les ignore, en grande partie, s'il ne les a pas sollicitées | 1 |  |
|  | (2) |  |
| Le clinicien permet au patient de poser des questions supplémentaires mais ne l'encourage pas à en poser d'autres et y répond sans entrer dans les détails | 3 |  |
|  | (4) |  |
| Le clinicien encourage ouvertement le patient à poser des questions supplémentaires (et y répond avec une certaine précision). | 5 |  |
| J. Etablit un plan de suivi clair | | |
| Le clinicien n’évoque aucun plan de suivi | 1 |  |
|  | (2) |  |
| Le clinicien évoque un suivi, mais ne fait pas de plan précis | 3 |  |
|  | (4) |  |
| Le clinicien établit un plan de suivi précis et spécifique de la consultation | 5 |  |
| *TOTAL (/115)* |  | |
