# Supplementary figures and images for: Cross-cultural adaptation of the 4-Habits Coding Scheme into French to assess physician communication skills
Source: PLoS One. 2020 Apr 16;15(4):e0230672. doi: 10.1371/journal.pone.0230672 (PMC7161987; doi:10.1371/journal.pone.0230672)

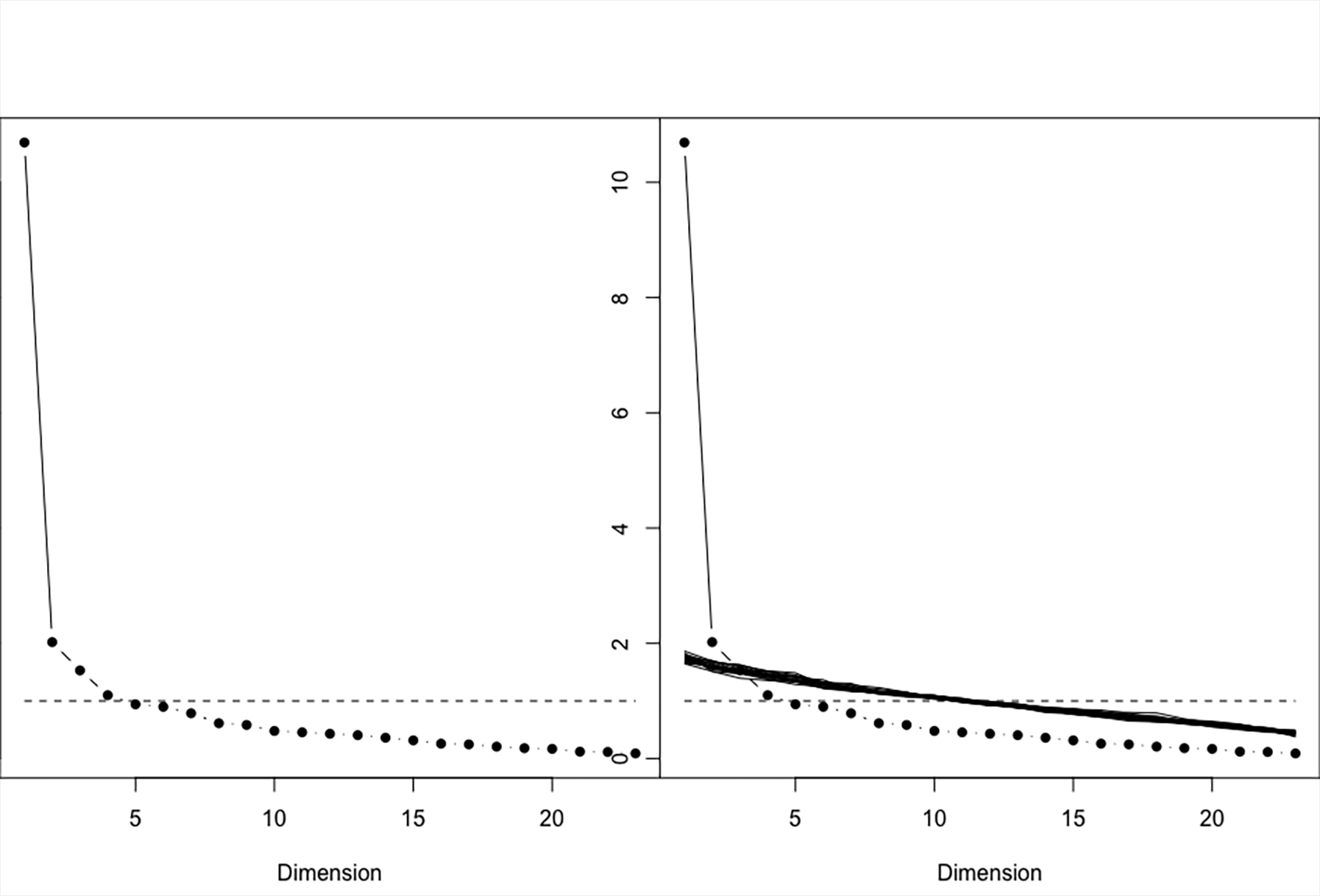

Supplement: S2 Appendix — (TIF) [file pone.0230672.s002.tif]
